# Yoga as a Holistic Treatment for Chronic Illnesses: Minimizing Adverse Events and Safety Concerns

**DOI:** 10.3389/fpsyg.2019.00661

**Published:** 2019-04-02

**Authors:** Shirley Telles, Sachin Kumar Sharma, Niranjan Kala, Acharya Balkrishna

**Affiliations:** Patanjali Research Foundation, Haridwar, Uttarakhand, India

**Keywords:** yoga, chronic illness, contraindications, adverse effects, safety

## Introduction

Yoga is widely practiced for its health benefits (Alter, 2004; Singleton, 2010), especially for chronic non-communicable diseases (Holte and Millis, 2013). The Consortium of Academic Health Centers for Integrative Medicine located at Michigan, U.S. teaches yoga in most of its branches (Holte and Millis, 2013; https://imconsortium.org/)^1^. Yoga is one of the top ten complementary health practices used by adults in the U.S. where 45 percent of this population have at least one chronic illness (Wu and Green, 2000). Two meta-analyses were carried out to examine yoga in the context of stroke (Lawrence et al., 2017; Thayabaranathan et al., 2017). The authors of the meta-analyses concluded that yoga is effective but did not report any adverse event associated with yoga practice (Lawrence et al., 2017; Thayabaranathan et al., 2017). Despite this, safety is important during stroke rehabilitation, especially for practices which involve maintaining balance. Similarly, we found that in meta-analyses of yoga used for multiple sclerosis or cardiac disease there was a lack of information related to the safety of yoga interventions as adverse events related to yoga practice were not usually mentioned (Cramer et al., 2014, 2015a). Apart from this, when safety related data were reported there were no adverse events (Cramer et al., 2014, 2015b). Safety issues in yoga practice apply to all chronic illnesses.

### The Need for Safety in Yoga Practice

An ancient *Hatha* yoga text gives importance to the method of practice, stating “….by the proper practice of *pranayama* (voluntarily regulated yoga breathing), all diseases are eradicated, whereas through the improper practice all diseases can arise” (*Hatha Yoga Pradipika, Circa* 1500 A.D., Chapter II Verse 16; Muktibodhananda, 1998).

William Broad (2012) attempted to highlight the adverse events which could occur with yoga practice in his book “*The Science of Yoga: The Risks and Rewards*” (Broad, 2012). This evoked a wide range of responses, especially from those who have benefitted from yoga practice. However the adverse events related to yoga cannot be discounted.

Hence this opinion article has two aims. (i) The first aim is to cite published examples of adverse events occurring from yoga practice due to: (a) an unusually long duration of yoga practice, (b) practice of a yoga technique more frequently than is recommended, (c) excess strain on a specific joint during yoga practice, or (d) ignoring any health condition which would be a contraindication for yoga practice. (ii) The second aim is to suggest recommendations to improve safety and reduce adverse events related to yoga practice as therapy.

### Search Strategy for Published Reports on Adverse Events Related to Yoga Practice

The search strategy was carried out in two stages. (i) The authors identified common reasons which could lead to an adverse event due to yoga practice, based on (a) the authors' experience with yoga practitioners and (b) their awareness of adverse events resulting from yoga reported by conventionally trained medical practitioners. This information was in the form of oral reports from 1997 to 2018. The most common reasons for adverse events were (a) practicing yoga for an extra duration, (b) practicing yoga more frequently than is recommended, (c) excess strain on a specific joint during yoga practice and (d) adverse event related to a prior health condition. (ii) Two researchers independently searched PubMed abstracts from 1970 to 2018 for examples to demonstrate the four points mentioned above. Four examples were selected as the most appropriate and are described in the manuscript.

### Published Reports on Examples of the Four Causes of Adverse Events Following Yoga Practice

#### An Example of Practicing Yoga for an Extra Duration

A 22 year old male, healthy college student who practiced the diamond pose (*vajrasana*, in Sanskrit) for 6 h a day for 2 months reported an abnormal gait due to foot drop (Chusid, 1971). The student had 18 months of experience in yoga. The subject recovered from foot drop after 9 weeks of discontinuing the posture. Practicing a yoga posture for 6 h a day is unusual; the recommended duration is 5–10 min for a beginner and not more than 30 min for an experienced practitioner (The divine life society^2^, http://yogaindepth.blogspot.com/p/detailed-description-of-yoga-asanas.html)^3^. In this case foot drop could be considered a consequence of practicing a yoga posture for a longer duration than is recommended.

#### An Example of Practicing Yoga More Frequently Than Is Recommended

In certain cases it may not be the duration but the frequency of the practice which was excessive. Regurgitative cleansing (*kunjal-kriya* in Sanskrit) involves voluntarily induced vomiting after drinking saline water on an “empty stomach” upto a point where the practitioner feels the urge to vomit (Saraswati, 2012). This yoga cleansing technique resulted in dental erosion in a 38 year old male who had practiced the technique every morning for 12 years (Meshramkar et al., 2007). While an ancient *Hatha* Yoga text describes *kunjal kriya* as useful to reduce digestive disorders, it is stated that the practice should be done once a week under the supervision of an experienced yoga teacher (Saraswati, 2012). Endogenous gastric acid enters the oral cavity during vomiting (José et al., 2008). The pH of gastric acid is approximately 1.2, which is below the critical value for demineralization of the teeth (José et al., 2008). This may explain the dental erosion in the 38 year old male in the study cited above, where a yoga technique was practiced more frequently than is recommended.

#### An Example of Excess Strain on a Specific Joint During Yoga Practice

Another factor which could be responsible for the adverse events following yoga practice include techniques which could either strain a joint or makes it unstable (Nagura et al., 2002). A cross-sectional study conducted in southern Thailand included 576 persons (276 females; 40 years or older) without any rheumatic diseases (Tangtrakulwanich et al., 2007). The aim was to correlate radiographic knee osteoarthritis with the habit of sitting on the floor for various activities, in sitting postures which resembled yoga postures as mentioned below. Those participants who reported squatting (similar to the chair pose or *uttakatasana*), side-knee bending (similar to the hero pose or *veerasana*), the lotus pose (*padmasana*) and total life time floor activities in highest tertile, showed a two time increased risk of osteoarthritis of the knee compared to those in the lowest tertile of exposure to floor seated activities (Tangtrakulwanich et al., 2007). These postures involve extreme flexion of the knee which causes excessively large contact stress on the knee joint (Dahlkvist et al., 1982; Nagura et al., 2002), which in turn causes cartilage damage and also acts as a precursor for degenerative diseases of the joint. Whether squatting is indeed harmful definitely needs thorough investigation, however the report cited above suggests the necessity for such studies and for precautions during yoga practice.

#### An Example of an Adverse Event Related to a Prior Health Condition

Another factor which could result in ill effects of yoga practice is an existing condition which may predispose the practitioner to deformity or illness (Cramer et al., 2013). A case study reported that sitting in the lotus pose during a meditation session resulted in a spontaneous supracondylar femoral fracture in a 58 year old Buddhist monk who was human immunodeficiency virus (HIV) positive (Pinto Neto et al., 2011). Patients with the HIV virus have compromised bone density due to the use of antiretroviral therapies which are associated with bone loss and fractures (Puthanakit et al., 2012). The lotus pose exerts stress on the supracondylar femur through upward force from the ankles and downward force from the knees. Stress on the compromised femur of the patient who was HIV positive could have caused the supracondylar fracture. This case report shows the importance of knowing if a person practicing yoga has any health condition which could make it dangerous for them to practice specific yoga techniques.

### Reporting of Adverse Events in Existing Trials

In contrast to the above mentioned reports on the adverse events associated with specific yoga practices there are a large number of studies on the benefits of yoga practice to improve physical and mental health (Hagen and Nayar, 2014; Jeter et al., 2015). Most of the studies which were conducted to assess the efficacy of yoga practice did not identify or report adverse events in the trials (Cramer et al., 2015b) In the meta-analyses of yoga used for stroke, multiple sclerosis and cardiac disease there was a lack of information about the safety of yoga practice, as adverse events were not usually mentioned (Cramer et al., 2014, 2015a; Lawrence et al., 2017; Thayabaranathan et al., 2017). This may be due to the fact that in such studies the yoga interventions were designed and delivered under the supervision of experienced yoga teachers (Cramer et al., 2015b) whereas in the four studies mentioned above on the adverse effects associated with yoga practice the subjects were either (i) practicing the yoga technique incorrectly, (ii) having precondition(s) related to the reported adverse effect, or (iii) were not aware that the particular yoga technique they were performing could worsen the precondition that they had. It could also be that adverse events were not reported or noted by the yoga teachers as they were not trained to do so.

### Precautions for Safe Yoga Practice

#### Descriptions From the Traditional Yoga Texts

Ancient *Hatha* yoga texts emphasize that yoga practices should be performed under the supervision of an able teacher stating “one should practice yoga as instructed by his guru” (*gurupadiūha-mārgheṇa yoghameva samabhyaset*, in Sanskrit; *Hatha Yoga Pradipika, Circa* 1500 A.D., Chapter I, Verse 14; Muktibodhananda, 1998). Hence though there is an increase in the number of studies reporting the efficacy of yoga to improve physical and mental health, the practice should be done with caution and under the supervision of an experienced yoga teacher.

#### Organizations Related to Providing Guidelines for Yoga Practice in India, the U.S. and Australia

There are known organizations to train yoga teachers and give them guidelines in different parts of the world. Examples are cited here from India, the U.S. and Australia. This is not all-inclusive as (i) there may be other organizations in India, U.S. and Australia and (ii) other countries also have similar organizations. Hence this is a non-representative description.

In India there are three main institutions which give guidelines for yoga practice. These are (i) the Morarji Desai National Institute of Yoga^4^ started in 1976 as the Central Research Institute of Yoga which continued as MDNIY from 1988, (ii) the Central Council for Research in Yoga and Naturopathy (Central Council for Research in Yoga & Naturopathy, www.ccryn.gov.in)^5^ and (iii) the Indian Yoga Association (Indian Yoga Association, http://www.yogaiya.in/about/)^6^. The CCRYN and the IYA were established in 1978 and 2008, respectively. The MDNIY and CCRYN are non-profit organizations funded by the Ministry of AYUSH, Government of India while the IYA is a non-profit, self-regulatory body approved by the Ministry of AYUSH and the Ministry of Health and Family Welfare, Government of India. The three organizations have no specific guidelines for reporting adverse events which occur during yoga practice.

In the U.S. there are the International Association of Yoga Therapists (IAYT, https://yogatherapy.health/about-iayt/)^7^ and Yoga Alliance (www.yogaalliance.org)^8^ which provide guidelines about teaching yoga. IAYT was established in 1989 while Yoga Alliance was started in 1997. The institutions are not-for-profit organizations which prepare national standards for training of yoga teachers in the United States and support research and education related to yoga as a therapy. These organizations do not have guidelines for adverse event reporting related to yoga practice. Also the National Center for Complementary and Integrative Health (Briggs, 2013) has mentioned safety concerns in yoga practice but there are no suggestions about reporting of adverse events due to yoga.

In Australia, the Australasian Association of Yoga Therapists (www.yogatherapy.org.au/)^9^ and Yoga Australia (www.yogaaustralia.org.au)^10^ are two associations which provide guidelines for yoga teachers and those who use yoga therapy. The associations were established in 1991 and 1999 respectively. They are not-for-profit organizations which were founded to bring yoga teachers from different traditions together and establish yoga therapy as a recognized, professional mode of treatment. Both these associations have no specific guidelines for reporting adverse events due to yoga practice.

### Recommendations for Safe Use of Yoga at Different Levels

It is recommended that (a) the duration and frequency of yoga practice, (b) the amount of strain on a specific joint during yoga practice, and (c) prior health conditions should be taken into account before starting yoga, to minimize adverse events associated with yoga practice. At various levels care should be taken to ensure that yoga practice is safe and to reduce the chance of adverse events. The recommendations for the safe use of yoga at different levels have been summarized in Table 1.

**Table 1:** Recommendations for safe use of yoga at different levels.

**Persons involved**	**Recommendations**
**YOGA PRACTITIONER**	
a. Yoga practitioner (a person with normal health who learns and practices yoga for his/her own physical, psychological, cognitive, social and spiritual wellbeing)	1. The person should be motivated to learn yoga. 2. Yoga practice should be under the supervision of a reliable and trained yoga instructor. 3. The yoga practitioner should view yoga instructors with respect, but should not be confused by concepts of a “*guru*” or the “need to surrender” which could lead to weakness, a dissolution of boundaries and hence exploitation (Khouri, 2018, https://www.yogitimes.com/article/yoga-business-teacher-student-boundaries-relationships-friendship). 4. Any earlier accident/injury or surgery should be mentioned to the yoga instructor. 5. The person should approach yoga for self-improvement without a sense of competiveness which could lead to going beyond his/her physical ability and hence resulting in injury.
b. Yoga practitioner (a person with a diagnosed health condition who learns and practices yoga primarily for the management of his/her health condition as well as his/her own physical, psychological, cognitive and spiritual wellbeing)	1. The person should give a detailed description of their medical condition to their yoga therapist. 2. The person should be clear about the objectives to practice yoga i.e., the practice of yoga to alleviate symptoms of a chronic illness and possibly to help manage the cause of the condition. 3. Yoga practitioner should have realistic expectations and not expect miraculous cures. 4. There should not be any attempt to modify the practice given to the person. For example if a person misses the yoga session on 1 day increasing the practice the next day may not be appropriate. 5. If the yoga practitioner notices any changes in their symptoms or their medication is altered by the physician, this should be reported to yoga therapist.
Yoga instructor (a person who has undergone training to teach yoga to healthy persons)	1. The person should be motivated to teach yoga. 2. The yoga instructor should be experienced in yoga practice and theory especially with an understanding about the exact way in which a yoga technique should be practiced as well as the duration, frequency and contraindications of the practice. This should be based on knowledge of traditional yoga texts (please see the footnote below)^a^ and the commentaries written on them. 3. A yoga instructor should be aware about the contraindications of yoga practices. 4. The yoga instructor should communicate with complete clarity about the method of practice and the contraindications of the practice. 5. All yoga instructors should have basic knowledge of physiology, functional anatomy and biomechanics. 6. The person should know the basics of first aid (e.g., treating minor injuries such as sprain). 7. A yoga instructor should be able to report an adverse event with sufficient accuracy (even with a diagram if necessary) to a relative of a yoga practitioner or even to a medical practitioner (if necessary).
Yoga therapist (a person who has undergone training to teach yoga for therapeutic benefits to patients)	1. A yoga therapist should have detailed case histories and knowledge of their patients. 2. A yoga therapist should be cautious attempting to treat patients who are weak, liable to fall, have poor balance or are otherwise “high risk cases.” 3. Yoga therapists require additional training compared to a yoga instructor. They need to know therapeutic yoga. in theory and practice and additional information about basic diagnostic methods and the disorders they may treat. 4. A yoga therapist should be able to record the pulse, blood pressure and body temperature accurately at the very least. 5. A yoga therapist needs knowledge of first aid and on-the-spot emergency treatments (e.g., cardiopulmonary resuscitation, managing choking, bleeding and fractures) as well as the contact details of a properly equipped hospital nearby. 6. If a person experiences any injury or harm during yoga, the yoga therapist should be able to report it to a doctor with details about the patient's medical history, the yoga practice (with a diagram if necessary) and the injury.
A doctor (a conventional medical practitioner)	A doctor who treats a patient with an injury or other discomfort believed to be arising from yoga should ask for clear details about the yoga practice performed, its duration, method and frequency. This information may be supplemented with diagrams or source material. Yoga practitioners should be clear that they are not being disloyal to their yoga teacher/school when giving this information.

a Hatha Yoga Pradeepika (Circa 1500 A.D.; Muktibodhananda, 1998), Gheranda Samhita (Circa 1700 A.D.; Saraswati, 2012), Patanjali's

*Yogasutras (Circa 900 B.C.; Ramdev, 2008) and Bhagwad Gita (Circa 400–600 B.C., Prabhupada, 2015)*.

There appears to be no organization responsible to record adverse events related to yoga. Hence an existing organization should take the initiative by having a centralized mechanism to report and track adverse events in a standard way. Organizations within a country and in different countries should come to a consensus so that this information is reported uniformly. This would be useful for yoga practitioners, yoga instructors and yoga therapists. It would also act as a resource for teaching and even to form policies.

## Author Contributions

ST compiled the manuscript. SS and NK assisted in compilation of the manuscript. AB assisted in compilation of the manuscript and provided the infrastructure.

### Conflict of Interest Statement

The authors declare that the research was conducted in the absence of any commercial or financial relationships that could be construed as a potential conflict of interest.

## Footnotes

^1^Academic Consortium for Integrative Medicine and Health [Internet]. *Academic Consortium for Integrative Medicine and Health* [cited 2019 Feb 15]. Available online at: https://imconsortium.org/

^2^Compression Design [Internet]. *How Long to Hold a Yoga Pose* [cited 2019 Mar 19]. Available online at: https://www.compressiondesign.com/how-long-to-hold-a-yoga-pose/

^3^Yoga Asanas in Depth [Internet]. *Detailed Description of Yoga Asanas Vajrasana* [cited 2018 Aug 27]. Available online at: http://yogaindepth.blogspot.com/p/detailed-description-of-yoga-asanas.html

^4^Morarji Desai National Institute of Yoga [Internet]. *Morarji Desai National Institute of Yoga: An Autonomous Organization Under Ministry of AYUSH Government of India*. Available online at: http://www.yogamdniy.nic.in/ (accessed February 15, 2019).

^5^Central Council for Research in Yoga and Naturopathy [internet]. *Central Council for Research in Yoga and Naturopathy: An Autonomous Body Under Ministry of AYUSH* [cited 2019 Feb 15]. Available online at: http://ccryn.gov.in/

^6^Indian Yoga Association [internet]. *About – Indian Yoga Association* [cited 2019 Feb 15]. Available online at: http://www.yogaiya.in/about/

^7^The International Association of Yoga Therapists [Internet]. *About IAYT* [cited 2019 Feb 15]. Available online at: https://www.iayt.org/page/AboutLanding

^8^Yoga Alliance [Internet]. *Yoga Alliance* [cited 2019 Feb 15]. Available online at: https://www.yogaalliance.org/

^9^The Australasian Association of Yoga Therapists [internet]. *The Australasian Association of Yoga Therapists* [cited 2019 Feb 15]. Available online at: http://yogatherapy.org.au/

^10^Yoga Australia [Internet]. *Yoga Australia* [cited 2019 Feb 15]. Available online at: https://www.yogaaustralia.org.au/
